# New statistical RI index allow to better track the dynamics of COVID-19 outbreak in Italy

**DOI:** 10.1038/s41598-020-79039-x

**Published:** 2020-12-22

**Authors:** Mariano Bizzarri, Mario Di Traglia, Alessandro Giuliani, Annarita Vestri, Valeria Fedeli, Alberto Prestininzi

**Affiliations:** 1 Systems Biology Group Lab, Department of Experimental Medicine, Sapienza University, Rome, Italy; 2 Department of Public Health and Infectious Diseases (Biostatistics Section), Sapienza University, Rome, Italy; 3 Istituto Superiore di Sanità, Environment and Health Department, Rome, Italy; 4 NHAZCA Srl, SpinOff; Earth Science Department-Sapienza University, Rome, Italy

**Keywords:** Immunology, Diseases, Health care, Pathogenesis, Signs and symptoms

## Abstract

COVID-19 pandemic in Italy displayed a spatial distribution that made the tracking of its time course quite difficult. The most relevant anomaly was the marked spatial heterogeneity of COVID-19 diffusion. Lombardia region accounted for around 60% of fatal cases (while hosting 15% of Italian population). Moreover, 86% of fatalities concentrated in four Northern Italy regions. The ‘explosive’ outbreak of COVID-19 in Lombardia at the very beginning of pandemic fatally biased the R-like statistics routinely used to control the disease dynamics. To (at least partially) overcome this bias, we propose a new index RI = dH/dI (daily derivative ratio of H and I, given H = Healed and I = Infected), corresponding to the ratio between healed and infected patients relative daily changes. The proposed index is less flawed than R by the uncertainty related to the estimated number of infected persons and allows to follow (and possibly forecast) epidemic dynamics in a largely model-independent way. To analyze the dynamics of the epidemic, starting from the beginning of the virus spreading—when data are insufficient to make an estimate by adopting SIR model—a "sigmoidal family with delay" logistic model was introduced. That approach allowed in estimating the epidemic peak using the few data gathered even before mid-March. Based on this analysis, the peak was correctly predicted to occur by end of April. Analytical methodology of the dynamics of the epidemic we are proposing herein aims to forecast the time and intensity of the epidemic peak (forward prediction), while allowing identifying the (more likely) beginning of the epidemic (backward prediction). In addition, we established a relationship between hospitalization in intensive care units (ICU) versus deaths daily rates by avoiding the necessity to rely on precise estimates of the infected fraction of the population The joint evolution of the above parameters over time allows for a trustworthy and unbiased estimation of the dynamics of the epidemic, allowing us to clearly detect the qualitatively different character of the ‘so-called’ second wave with respect to the previous epidemic peak.

## Introduction

After a novel strain of coronavirus—SARS-CoV-2—was identified in Wuhan (China)^1^, Italy resulted to be one among the most affected countries by COVID-19 epidemic^2^. Noticeably, despite the existence of an already established program to deal with such an outbreak, Italian government was taken by surprise. Despite warning coming from previous, disregarded epidemiological advices, clinical and administrative countermeasures lasted until March 2020 before to be adopted in a systematic way.

Predictive mathematical models are fundamental to forecast the course of the epidemic and to plan effective control strategies. Unfortunately, it is likely that unreliability of the preliminary data as well as inadequacy/lack of proper descriptive models played a relevant role in undermining political decisions of the Italian administration. For instance, the widely used Basic Reproduction Number (R) has been proven a severely biased estimate of epidemic spreading. Use of R in the popular press has led to misunderstandings and distortions of its meaning. In addition, R can be calculated by using many different mathematical models, and each of these can give a different estimate of R that require to be interpreted in the context of the chosen model^3^. Noticeably, R enables in assessing the extent of the infectious spread, but not the speed at which the infection grows. It should be better defined as a “retrospective” parameter strictly depending upon assumptions on the nature of epidemics spreading. As such, R is not a “predictive” marker and it has been widely criticized, especially in the context of the COVID-19 epidemic^4^.

Several models have been developed to describe the Sars-CoV-2 pandemic, by reframing the SEIR model^5^, using the discrete-time SIR model (which includes dead individuals)^6^, or a control-oriented SIR model that stresses the effects of delays and compares the outcomes of different containment policies^7^. Stochastic transmission models have been applied as well^8^.

The above-mentioned approaches stem from compartmental diffusion processes routinely used in physical chemistry and pharmacokinetics. SEIR stands for Susceptible-Exposed-Infected-Recovery that denote four states that each patient traverses going from susceptible (S), to the exposed (E) and infected (I) conditions ending up into the recovery (R), a compartment that collects both recovered and dead patients. The apparent oddity of putting into the same compartment healed and dead persons comes from the physical-chemistry origin of the model in which R compartment collects all the entities (e.g. molecules) arriving at the final step of the process (end products, excreted or adsorbed substances).

The addition of other compartments to the basic SEIR model can (at least in part) solve some problems of this approach. Notably, Pedersen and Meneghini^9^ introduced a ‘quarantined’ compartment ending up with a SIQR (Susceptible, Infectious, Quarantined, Recovered) model in which the Q compartment corresponds to the persons recognized as positive and then quarantined. These subjects must be considered no longer contagious, having been isolated from the rest of the population. The authors of the above mentioned paper^9^—are aware of the impossibility to model the actual number of infected persons due to the unknown number of unidentified, asymptomatic COVID-19 infected individuals. Therefore, the paper self-limits to modelling ‘the rate a with which these patients become *non-infectious’* ^9^ (emphasis added). This is the same philosophy we adopted when considering dH index (based upon the H (healed) compartment, see below). Healed patients can be considered to derive from Q compartment, having been quarantined and then released after three negative tests*.*

So far so good, but the problem (when the model is applied to real processes) is hidden in the statement ‘on average’, that embeds the physical condition of ‘ergodicity’. In simple terms, this condition implies stochasticity of the contacts and (as for any averaging process) the homogeneity of the process.

Both these conditions are violated in the Italian case. Data highlighted differences of one order of magnitude in both infection dimension and spreading rate between Lombardia (accounting for around 60% of total fatalities and infections) and the rest of Italy. We will not discuss here the possible causes of this discrepancy (that, even if less dramatic, involves the whole Northern Italy, in which 86% of the overall Italian fatalities have been recorded), as this task would require a specific clinical/epidemiological inquiry. Instead, we focus on the possible ways to get a sufficiently accurate estimate of the epidemic process when in presence of huge violation of SEIR model premises. A statistical index different from the SEIR-derived ones, could be very useful to assess the entity of an epidemic outbreak.

The need of a different statistical index is still more cogent in the Italian case, considering that the Lombardia outbreak happened earlier than observed in other regions, and administrative officials were asked to take decisions based on limited information, which – at that time – was mainly restricted to Lombardia singularity. This situation could be not so rare. Thus, the need of a more robust statistical index is not limited to the Italian situation. Indeed, during the initial stages of a highly contagious, viral infection spreading through the air, there is not enough information to predict epidemic temporal development using analytical models, but some useful hints could be extracted as “hidden” information from simple statistics based on preliminary data. This is why, before entering into the new proposed index derivation, it is worth checking if the epidemic data collected during the first months, affected by both uncertainty and heterogeneity biases, do allow for a reliable forecasting by adopting less demanding models, with respect to SEIR ones. We achieved that goal by means of the Verhulst-Pearle logistic model (sigmoid), which was able to predict the occurrence of the peak of infection, when fed with very early data. We estimated the parameters by regression methods, applying the geometric mean as a function of truly infected individuals. The initial condition for the Bernoulli differential equation was determined applying the backpropagation method^10^.

Having demonstrated, by logistic approach, the presence of relevant (even if hidden) information on epidemic dynamics in the actual observational data, we went on to the second analytical phase. This step consisted in the derivation of a novel index (RI). This index stems from the splitting of the R compartment into healed and dead persons, ending up into the dH/dI (RI) index with dH and dI being the daily (day(i)-day(i-1)) differential of healed and infected people, respectively. This choice allowed us to detect a clear tipping point in epidemic dynamics in Italy (without considering data from Lombardia), thus recognizing the onset of the decline in the epidemic cycle. Such tipping point has emerged only later in Lombardia, and this finding is consistent with the anomalous dynamics of the epidemic in that region.

We complemented the investigation by a concomitant estimate of the clinical severity of COVID-19 disease, which should allow grasping both the potential lethality of COVID-19 and the effectiveness of hospital care in sustaining the “medical impact” of the epidemic. Underestimation of asymptomatic (positive) COVID-19 bearing subjects and uncertainty in detecting the true number of infected people, due to limitation in both availability and reliability of oral-pharyngeal swab data during the first months of the epidemic, severely undermined the trustworthiness of raw lethality data (Case Fatality Ratio, CFR, corresponding to the death/infected ratio), mostly because true incidence resulted underestimated. CFR values relative to different regions convey information on both age distribution (COVID-19 fatal cases are almost completely restricted to patients > 70 years old) and efficiency of public health systems^11^. In fact, Italy has the oldest population in Europe and the second oldest population in the world, after Japan. COVID-19 has shown a strong dependence on age due to the severity of the infection and the risk of death^11^. However, CFR in Japan is much lower (3.2%) than that recorded in Italy (8,8%, October 2020), despite their similarities. Therefore, to obtain a more reliable appraisal of the clinical evolution, we looked for a new epidemic marker, independent from the potentially flawed estimate of the actual number of infected individuals. Thus, we focused on the daily number of accesses of COVID-19 patients to the intensive care unit (ICU). Herein, we show that admittance rate to the intensive care units shows a very high linear correlation with the number of casualties so that the slope of this relationship is a good faith estimate of the effectiveness of hospital care as well as an unbiased index of the clinical evolution of the epidemic.

The shared rationale of the above-mentioned strategies is the ‘blessing of delay’: in order a patient is actually registered as ‘healed’ it is necessary to wait for three negative tests; this creates a time delay with respect to the ascertainment of infection cases. This delay (together with the latent but unescapable correlation between infected and healed frequencies) makes the ratio between daily changes in healed and infected compartments a proxy of the time derivative of infection dynamics only marginally affected by infected number uncertainty. Similar considerations hold for intensive care unit hospitalization and fatalities that in turn do not include any infection number estimate.

## Methods

### RI index

**Figure 1:**
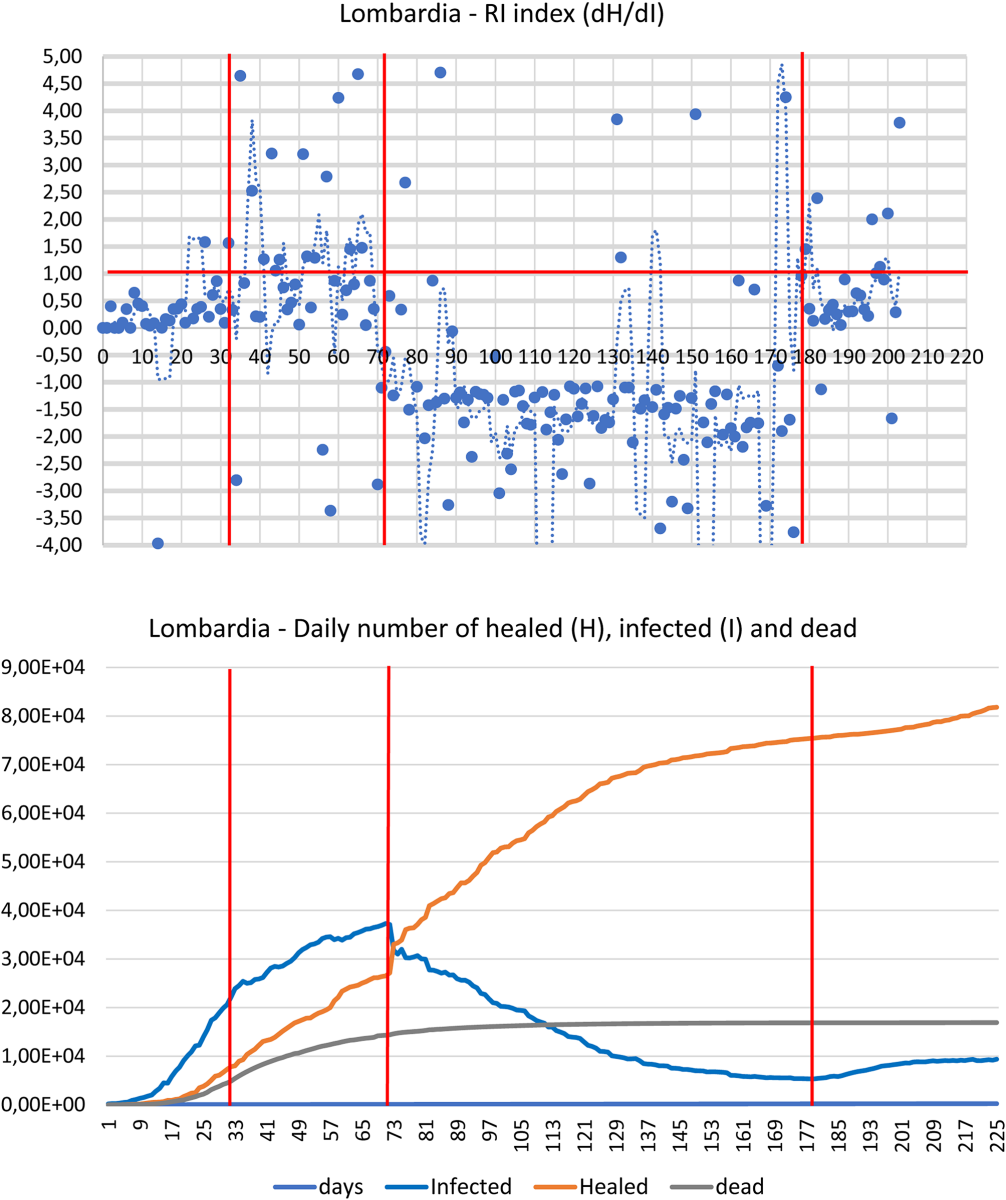

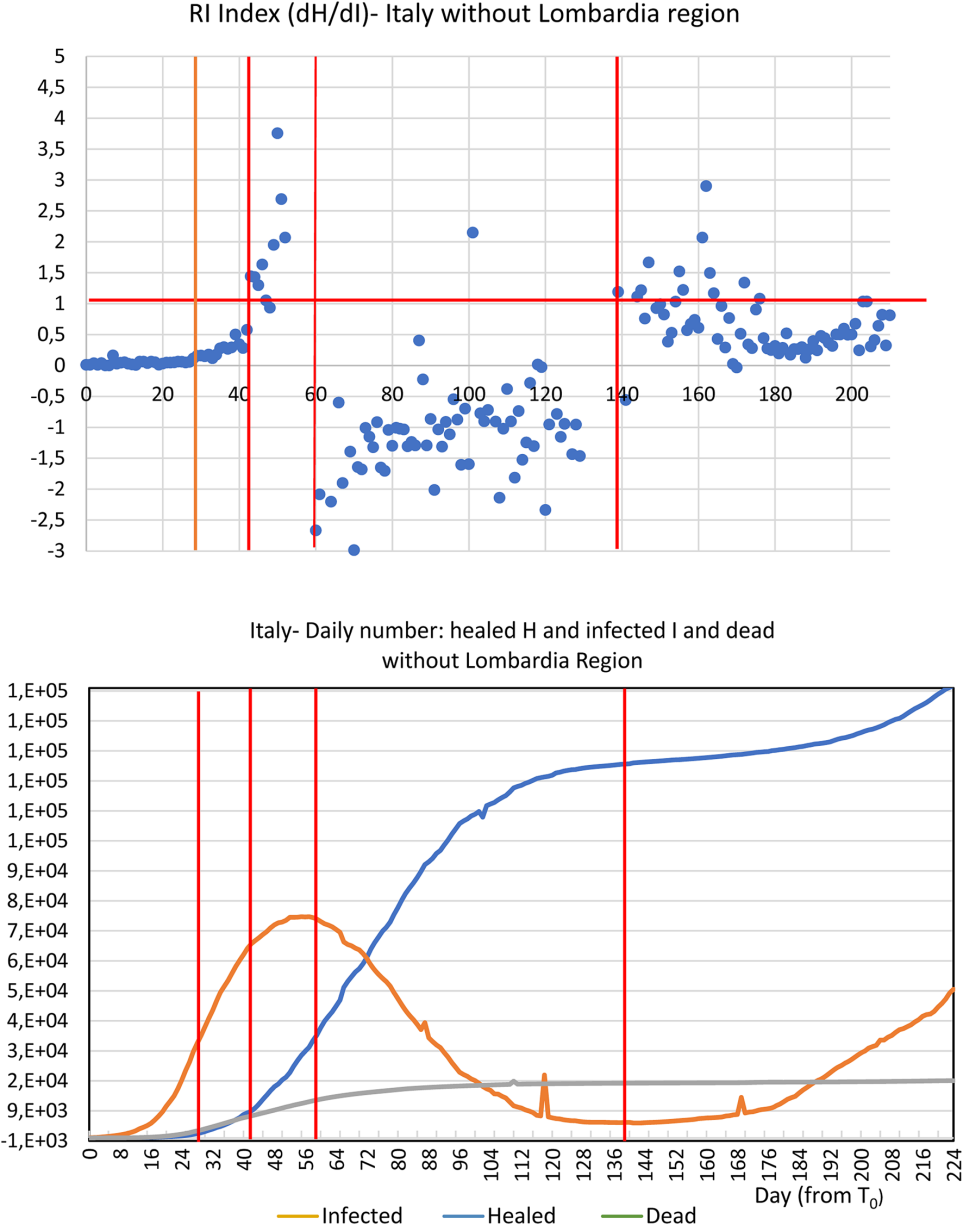

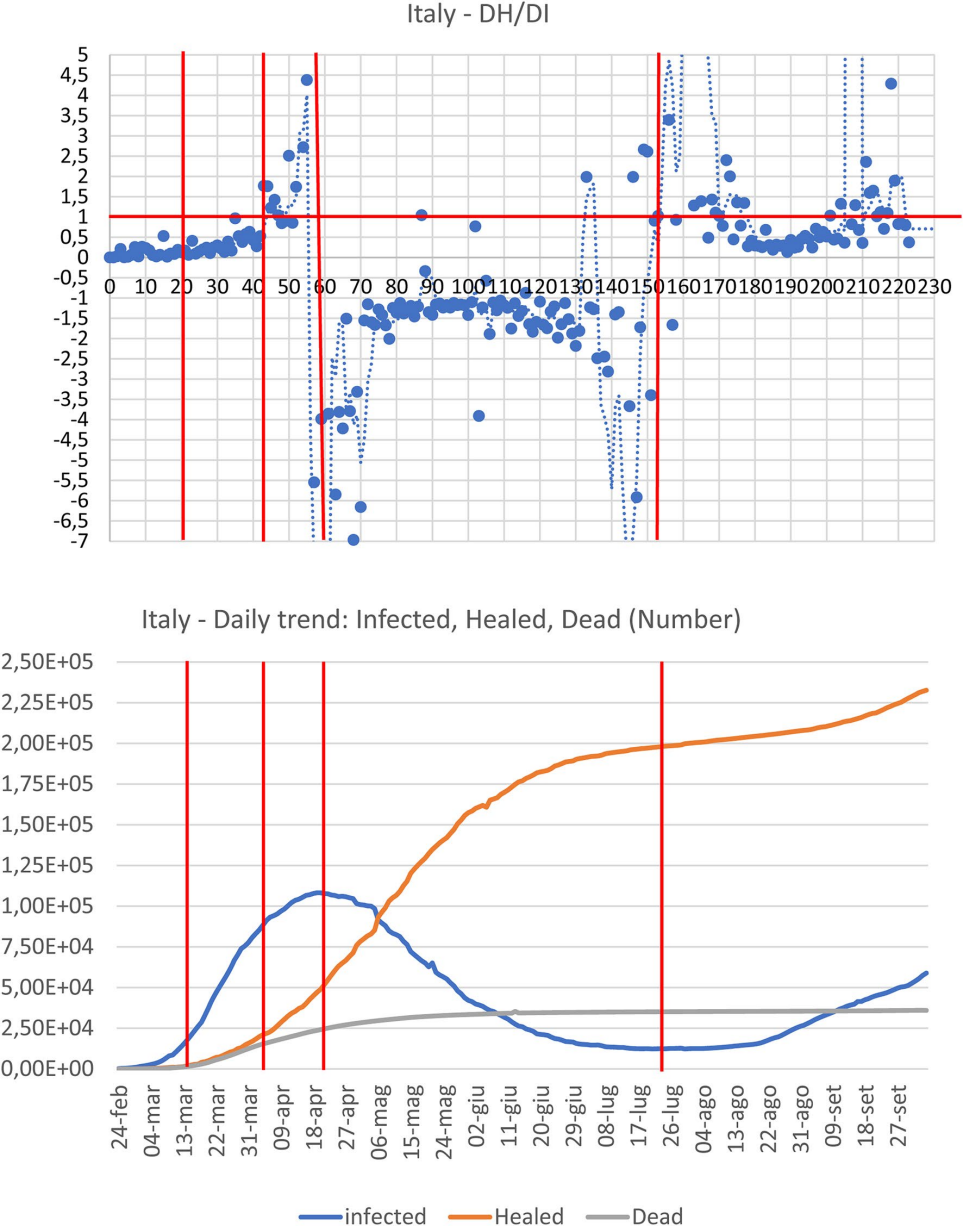
The figure reports for both Lombardia, Italy without Lombardia, and Italy the dH/dI (RI) index daily variation **(A,C,E)**, the cumulative Dead and Healed count together with the Infected daily number. and the dH and dI daily variation **(B,D,F)**. The graphs are subdivided into five temporal windows corresponding to the Beginning, Rising, Plateau and ‘Second Wave’ phases respectively (see text). The presence of a ‘tipping point’ of epidemic is evident around 20 of April in all the different representations as intersection of ‘rising’ healed and ‘declining’ infected curves. The only (partial) exception is the Lombardia dH vs. dI plot were the tipping point is blurred between April and May. This behaviour is consistent to the much higher variability of RI index of Lombardia, pointing to the presence of ‘super-spreaders’ hubs.

Analyses and calculations were based on publicly available official data, provided by the Italian Health Administration, recorded daily for each Italian province (in Italy each region is further divided into provinces)^12^. The time window taken into consideration went from the end of February to 12 May 2020. The division of this time window into "fields" derives both from the need to compare different regional entities and to be consistent with different stages of epidemic curve. Accordingly, the average value of the RI (dH/dI) index was calculated from the healed and infected curves within each field (Table 1, Fig. 1). The fields correspond to T0–T1 = starting point, with a very low number of infected and almost no healed patients. T1–T2 = initial exponential increase in the epidemic phase, featuring a marginal number of infected compared to healed patients. T2–T3 plateau phase, when the curve of infected patients shows an initial trend toward reduction with simultaneous exponential increase in the number of healed individuals. T3–T4 = declining phase, with RI index going into negative territory due to the declining values of infected and consequent negative values of the denominator going hand-in-hand with an increase in the healed. > T4: the new phase, the so called ‘second wave’ with the appearance of a new dynamical regime.

**Table 1:** Comparison between R and RI indexes.

Italy						Lombardia region					Italy—without Lombardia region				
Field	Time window		RI	σ/RI	Rt	Field	Time window	RI	σ/RI	Rt	Field	Time window	RI	σ/RI	Rt
T_0_–T_1_	February 24–March 13		0.11	1.24	1.12	T_0_–T_1_	February 24–March 31	0.34	4.01	1.40	T_0_–T_1_	February 24–March 22	0.03	0.43	1.26
T_1_–T_2_	March 14–April 6		0.30	0.73	0.94	T_1_–T_2_	Aril 1–May 3	1.10	2.05	1.01	T_1_–T_2_	March 23–April 6	0.24	0.57	0.99
T_2_–T_3_	7 April–19 April		2.21	0.81	0.94	T_2_–T_3_	May 4–August 18	− 1.71	2.59	1.14	T_2_–T_3_	April 7–April 19	6.07	1.65	0.98
T_3_–T_4_	April 20–July 24		− 2.60	1.15	1.03	> T_3_	> August 19	1.31	3.59	1.11	T_3_–T_4_	April 20–July 10	− 1.16	4.99	0.94
> T_4_	> July 25		0.89	2.28	1.05						> T_4_	> July 10	0.78	4.22	1.07

This table compares RI and Rt, within the time windows (Tn-Tn-1), defined for the three cases examined: Italy, Lombardy and Italy without Lombardy. It clearly emerges that the RI index records the acceleration and deceleration of the epidemic curve, both over time and in the different areas of the examined Italian territory. In the first phase, this acceleration seems to be connected to the initial imbalance between the newly infected and the healed, with an increase in the number of infected but with a reduced increase in the healed. In this case RI assumes low values close to zero. In the same phase, however, the R (t), while recording high values of the new positives, does not show the rapid evolution of the healed, as happens with RI, which assumes increasing values assuming values > 1. The fast evolution of the pandemic curve, with a rapid reduction of the new infected, is denounced by the values by RI, which assumes values < 0. In the later stages, the recovered play a dominant role and, therefore, the RI index drastically increases while Rt decreases due to the overcoming of the epidemic peak. The phases following the peak are described by the two indices in a different way as Rt remains substantially stable but RI shows a greater sensitivity to changes in the infected.

It is worth remarking that the infected, dead, and healed compartments are non-overlapping, as each patient is considered healed only after three negative PCR results performed across a period of three weeks. The robustness to the uncertainty of the infected number of the proposed index derives from both the use of the daily differential (instead of the total number of infected patients), and the long delay between the infection and the declaration of "complete recovery", which is obtained only after three PCR negative test.

### Logistic model

**Figure 2:**
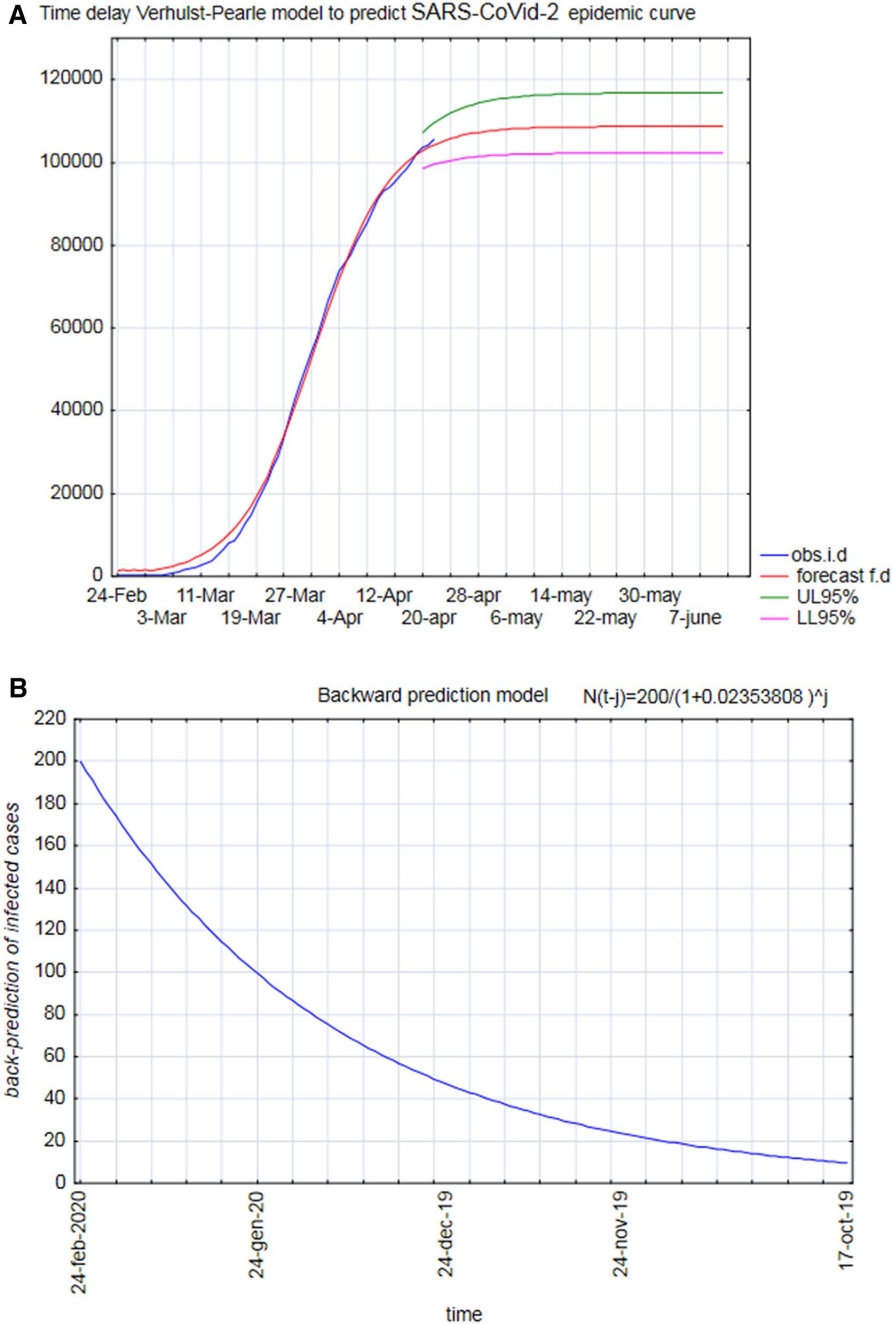
The figure reports the logistic model of epidemic cycle built upon the end of March 2020 data, with the indication of backward and forward prediction confidence intervals (**A**). **(B)** Focuses on backward prediction pointing to the very beginning of epidemic in October 2019.

In the initial stages of the infection, there is not enough information to predict epidemic temporal development using too demanding models. Thus, we approached the forecast of time and intensity of the peak of infection through the Verhulst-Pearle sigmoid model (Fig. 2). The derivative of the sigmoid indicates (in the peak) the inflection point of the epidemic curve and describes the change of speed in the spread of the epidemic. This change, in Italy, occurred around the end of March. The parameters of the model were estimated by applying the geometric mean as a function of currently infected. The parameters, *a* and *b*, of this function are estimated by regression methods. The initial condition for the Bernoulli differential equation, N(0) = 224 and N(l) = 8514, (where l is the time delay) was determined applying the back-propagation method^10^.

### Regression analyses and trajectories in the phase space

In order to get an idea of the epidemic dynamics without relying at all on estimate of the number of infected persons, most potentially biased element of the entire picture, we performed a simple linear regression (for both Lombardia and the rest of Italy) linking the number of patients in intensive care units (ICU) and dead.

**Figure 3:**
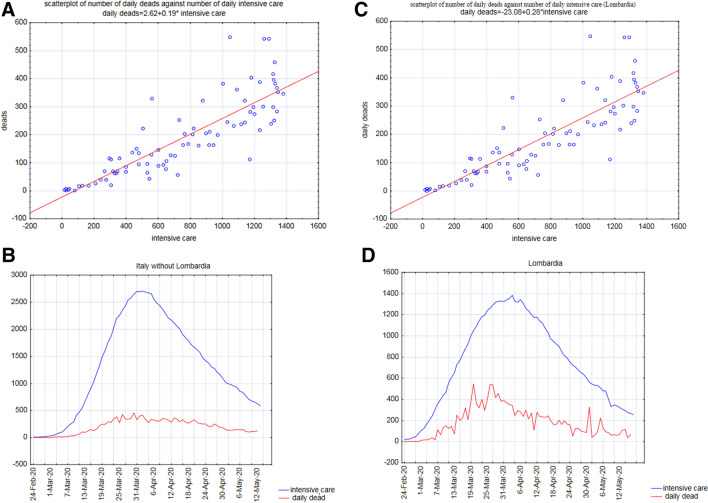
**(A,B)** Report the regression line linking the number of patients in intensive care units (ICUs) and the number of dead on a day-by-day basis for both Lombardia and Italy. **(C,D)** Report the daily variation of dead and ICUs for Lombardia and Italy, respectively. It is worth noting both dead and ICUs follow almost perfectly the epidemic course.

**Figure 4:**
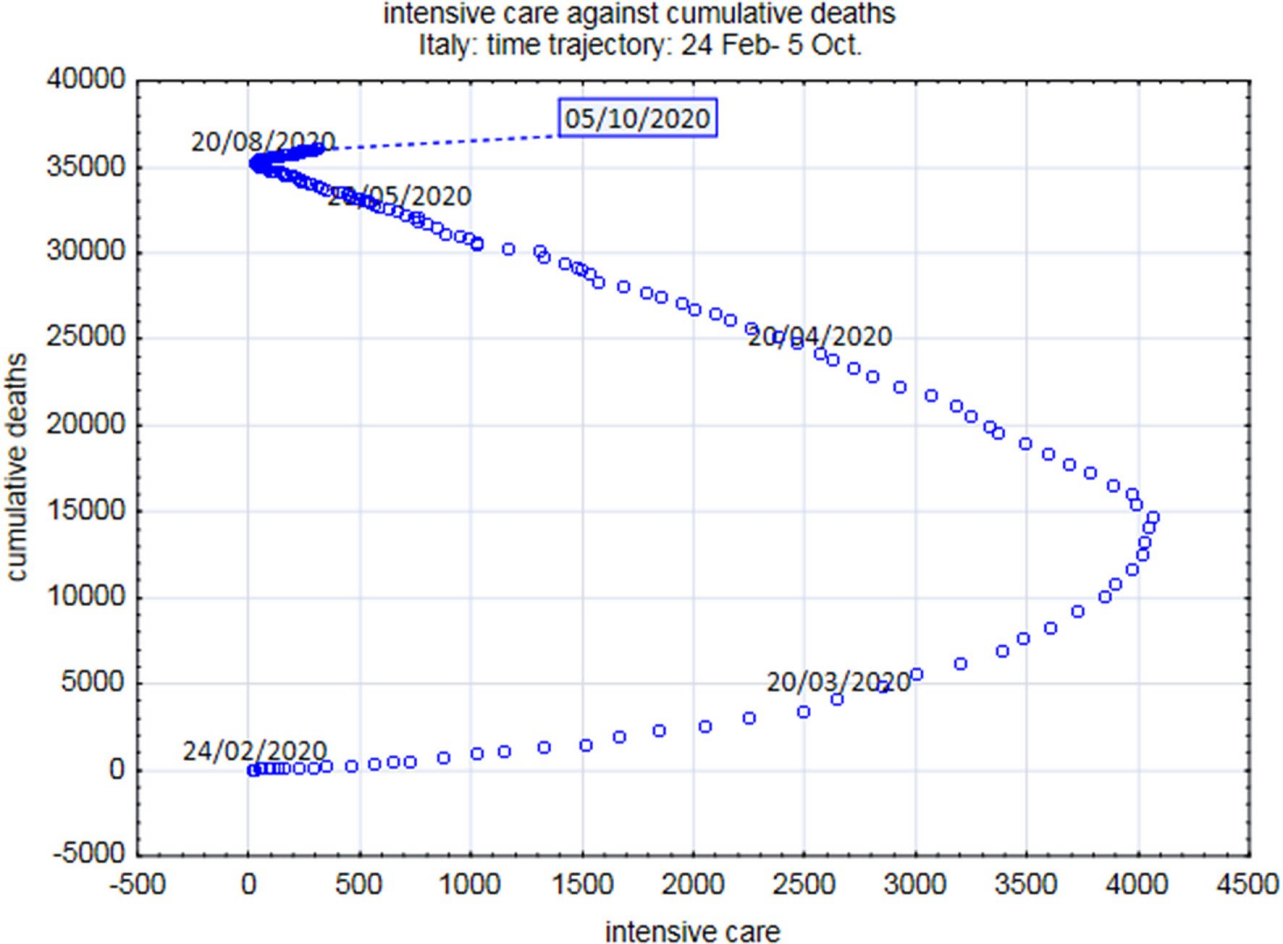
The figure reports the trajectory in the ICU/Cumulative Death phase space of the epidemic. While the dead count exactly follows the epidemic course until 20 June 2020, starting from that date, the dead count is decoupled by the rising of infected number. It should be stressed that, from June 20, the death rate is dramatically slowed down, mirroring the contemporary reduction in the accesses to ICUs.

The obtained regressions were driven by the relation with the time course of epidemic of both ICU and Dead number , as evident from both the ‘bell shape’ curves of ICU and Dead during epidemics cycle (Fig. 3) and the phase space portrait reporting the number of infected and dead trajectories (Fig. 4). This portrait allowed us to catch the drastic difference between the February–June epidemic cycle and the so called ‘second wave’ where the number of fatalities is drastically decoupled by the epidemic dynamics.

## Results

### Epidemic trend in Italy and Lombardia region

Table 1 shows the dynamics of the RI index (dH/dI, calculated as the average value at different time intervals both in Lombardia and in the rest of Italy). The different time intervals (“fields”) correspond to the peculiar behaviour of infectious dynamics, described in the methods section. It is worth noting that, beside the much higher values of cases in Lombardia, the quality and timing of the different phases are superimposable to Italy, so allowing for a convergence estimation of the global dynamics of the pandemics (that in turn is not possible by means of R index). On the quantitative side instead, the RI is three times higher in Lombardia than in the rest of Italy in the plateau phase (6.1 vs. 2.2) consistently with the higher spread of the disease in this region. It is worth noting how R index displays a very high variance that (especially in the first phases of epidemic cycle with e very low number of tests) does not allow any reliable estimate of infection spreading rate. In the case of Lombardia versus Italy, the huge variability of R can probably be ascribed to “super-spreaders” infected individuals, which act by “destroying” the supposed ergodicity of the studied phenomenon. What should be stressed is that R index cannot detect the reaching of 'tipping point' of epidemic and is unable to recognize the transition between different phases (Table 1). It is worth of notice that the new RI index we are proposing here can specifically help in recognizing such tipping points, thus enabling to follow the epidemic evolution and how/when the epidemic trend assumes an opposite direction.

Raw data have been provided by daily sampling made by Italian Health Administration and are affected by biases due to non random sampling and test uncertainty (especially important in the first phases of the epidemic).

As can be observed in Fig. 1A,C,E, it is worth noting how RI value reaches a ‘tipping point’ marking a phase transition^13^ of the dynamics at Field 3 (T2–T3). The healed daily variation overcomes the infection rate, and the dead daily number decreases while healed curve (after intersecting the dead one) drastically increases (Fig. 1B,D,F). Here RI has a huge variance, another signature of phase transition^13^. In this time interval, the infection rate reaches its plateau while healed curve displays a neat acceleration. The plateau phase is in turn coincident with the prediction made by logistic curve based on a completely independent approach.

All in all, the proposed index tells us of the reaching of a tipping-point at day 45 from the beginning of analysis from there a rapidly declining phase starts with the end of epidemic located around 20 of June (as will be more evident by subsequent analyses of dead/infected trajectories).

### Logistic model for backward and forward predictions

The Logistic model originated from modelling attempts of population growth in ecology as an improvement on the Malthus population model, the original (1838) Verhulst equation being: dQ dt = rQ(1 − Q K ) where Q, r and K indicate the population size, intrinsic growth rate and maximum population size that the environment could carry, respectively. dQ/dt represents the growth of the population. r and K are constants numbers, and the value of Q derives with time to produce an S-shaped curve in this Logistic equation. In our application (being Q the number of cases) we inserted as constant values the number of infected at February 24 and March 16 (see text) so doing, we restricted the problem to a single parameter estimation while the form of the logistic pushes the model toward the S-shape curve. This shape has a deep motivation in the case of epidemics that can be summarized as: whatever the real number of cases, the actual infectivity of the agent, in the very first phase of the outbreak, the initial number of people infected is small, so the number of infections slowly increases. When the infection base reached a certain proportion, the epidemic situation has an exponential growth trend, and then (for both the existence of a finite number of susceptible people and government’s regulation and restrictions) the contagion rate goes down, finally reaching a plateau. The key point is the time at which the curve turns, i.e., when rapid increases in the number of cases are replaced by slow increases. Because this inflection point specifies the point at which the daily number of cases (again, no need to estimate the actual number of cases, the daily increase is sufficient) increases to a maximum, this moment marks a critical turning point at which transmission of the disease begins to decline. As long as the data include this key point and the time interval shortly thereafter, the curve fitting and prediction of the future number of cases will be fairly accurate^14^. To make a long story short, logistic model drastically weakens the ergodicity/homogeneity problem thanks to its very basic requirements**:** finding a S-shape trend of daily increases. The prediction ability of the model is then checked outside the interpolation window. It is worth noting that the same logistic strategy adopted by Zhang et al. (2020) on different countries predicts the reach of a global peak in October 2020. In any case the logistic model demonstrated to be good in epidemics prediction in other instances^15,16^.

From the above considerations descends that the logistic model assumes that the rate of change in the level of infected over time is a decreasing function of the level itself (usually a linear function with parameters α and β. Their ratio (*α N(t*)/*N(t* + *h*)*β*) indicates the peak, that is: how infected there are during the maximum period of the epidemic curve, (epidemic peak). The equation that expresses the dynamics of the rates, obtained from the observed data is thus:

$$R\left( t \right) = \alpha + \beta *I\left( {t - h} \right) \, + \varepsilon ; \, (\beta \;{\text{negative}})$$

The estimated equation is:

$$R\left( t \right) = \, 0.1466 \, + \, 0.00005173*I\left( {t - h} \right) \, + \varepsilon$$

In the case of forward forecasting, the model relies upon the following estimated equation:

$$N\left( t \right) \, = \, \left( {{8514}*a} \right)/\left( {exp\left( { - a*t} \right) \, + b*{224}} \right)$$

where, *a* and *b* are the estimate of *α* and *β.*

Being N (t) is the number of infected individuals at time t, 224 is the number of infected peoples on February 24, and 8514 the infected peoples at March 16.

The model shows a delay effect (autocorrelation) of 18–22 days in the epidemic curve (224 = number of infected peoples on February 24 and 8514 the infected peoples at March 16. Model fitting was based on the data relative to the interval of time February 24 –March 16, and the coefficients (with 95% confidence limits) were:

*a* = 0.1466 (0.1401, 0.1531); *b* = 5.173e−05 (4.605e−05, 5.741e−05).

Goodness of adaptation:

Square R: 0.9307; adjusted square R: 0.9294; RMSE: 1.057e + 04.

The model fitted with the data gathered at mid-March and was able to correctly predict the peak of the epidemic at the end of April (at April 20, the predicted and observed *N*(*t*) was respectively: 107,715 and 108,237). The confidence interval, (1 − α) = 0.95, indicates the fluctuation of the real amount of daily infected people during the peak approximatively (Ll = 102,000, Ul = 112,000). The real data was around 108.000 infected people (a number close to that predicted by the model).

The confidence interval (CI) for the estimated time t_p_ is obtained by the logistic inverse formula. By substituting in the inverse function, the extremes of the confidence interval for α and β, we obtain the confidence interval for the time in which the epidemic peak *t*_*p*_ is predicted.

With a 95% significance level, the model predicted that the peak would occur between the 50th and 64th day starting from February 24th. This range includes the days from April 13 to April 27, 2020. The point forecast of the peak occurred on April 20. On that day, in fact, the trend inversion of the epidemic curve took place. Exactly, as the model had predicted. In conclusion, indicating with t_p_ the day of the epidemic peak, we have:

$$\Pr \left\{ {} \right.t_{p} \in [13april;\left. {27april]} \right\} = 0.95$$

### Backward prediction model

The first available data on the number of daily infected in Italy was available on February 24, 2020. In the first 10 days of registration, epidemiological data resulted highly “unstable” and untrustworthy. Nevertheless, they can still capture the dynamics of the phenomenon. To eliminate excessive fluctuations, a four terms moving median was applied to the data. Furthermore, on the levelled series, the geometric mean of the rates of change was calculated. This average is 0.02354 (net rate). Finding the evolutionary dynamics of the number of infected, at the beginning of the exponential branch and coming from the asymptotic branch, the formula of compound capitalization is applied. We indicate with t the day February 24 and with j the number of days before t. The daily infected peoples at time t − j is given as follows:

$$N\left( {t - j} \right) \, = \, N\left( t \right)/(1 \, + a)^{j}$$

where:

$$N\left( {t - j} \right) = 202/\left( {1 + 0.02353808} \right)^{j}$$

*N*(*t*) is the levelled number of infected at day t, alpha is the geometric mean of the in the first 10 days and j is the numbers of days before t.

For j = 130, we get N (t − j) = 10; from February 24, subtracting 130 days we end up at mid-October. This unexpected finding provides an indirect confirmation about the hypothesis already formulated based on the estimate of genetic distances among different Sars-CoV2 isolated strains^17^. Noticeably, these findings strongly disagree with the official reconstruction of the epidemic onset, which posits that the first infection cases could be observed at the beginning of January 2020 only.

### Clinical severity estimation and phase space trajectories

The aforementioned Japan/Italy paradox, where two nations with largely super-imposable demographic structures show huge differences in lethality trends, asks for something different from the usual lethality indexes. We decide to remove the principal cause of uncertainty, i.e. the rate of infected people, by focusing on different observables. That goal was achieved by relying on the relation between the number of accesses in intensive care units (ICU) and the number of deaths. Both these variables are known with no (or very little) uncertainty and they give an immediate estimate of the severity of the disease (Fig. 3A,B).

Figure 3C,D reports the evolution of ICU as well as the COVID-19-related fatalities (recorded daily in Italy), excluding (Fig. 3C) and including Lombardia data (Fig. 3D). Figure 3C shows the parallel behavior of the number of accesses to intensive care and the number of deaths for the entire country. The two parameters are strictly correlated (Pearson r = 0.94), and the slope of the curve is equal to 0.19 pointing to an 81% of success for intensive care. This relation points to a constant ‘clinical relevance’ of the disease throughout the entire time course.

The figure refers to the same day for both death numbers and ICUs and is worth noting that the fit comes from the shared trend of the two variables both registering the same epidemic spread. As a matter of fact, the ‘lagged correlations’ (i.e. the correlations between number of deaths and ICUs, three, four, five and six days before) displayed a significantly lower correlation coefficient. The fact that ICUs-Fatalities relation is driven by the epidemic dynamics (the relation practically disappears when lagged correlations are computed) tells us that this relation can be considered as a proxy of the epidemic dynamics more than a purely clinical descriptor. Figure 3C,D describe the simultaneous evolution over time of the two parameters—ICUs and death—evidencing how the rise and the subsequent decline of the epidemic can aptly be depicted with the help of these observables. Moreover, the trend reported in Fig. 3C,D clearly show the superimposable ‘bell shape’ curves for both Lombardia and Italy, making evident the ability of both ICUs and Deaths to be reliable markers of the epidemic cycle. Keeping in mind that fatalities have a variable delay from hospitalization, the ICUs-Dead number function is equivalent to a temporal differential rate of infection scaled by a largely constant intrinsic fatality rate. At odds with other strategies, the ICUs-Dead number function does not imply any direct infection estimate.

Figure 3B refers to Lombardia, and again we observe a strict relation between the number of daily accesses to intensive care units and deaths. It is worth noting that while for the rest of Italy the slope was 0.19, the slope relative to Lombardia is quite higher (0.28) pointing to a decreased success of care dropping from 81 to 72%. By a closer look at Fig. 3B is evident how the right part of the plot (corresponding to the peak of the epidemic with higher number of both fatalities and intensive care accesses) has a higher displacement of observations from the regression line with a tendency to score a higher number of fatalities.

This asymmetric displacement could be the image considering the initial overcrowding of the hospitals in Lombardia that first faced the initial impact of the epidemic. In any case, the relation between hospital accesses and deaths can be considered as a useful parameter for monitoring disease severity. The corollary of this fact is that the number of healed individuals properly reflects the epidemic evolution in a clinically reasonable way, allowing overcoming bias provided by uncorrected data collection about incidence and lethality. Accesses to critical care units reach a peak at mid-March and then regularly decreases, reaching in these days (July 2020) very limited numbers (< 50 patients in respect to ~ 4000 at mid March). We hypothesize that this behaviour reflects the improvement in treatment of patients that are followed at home or in standard hospital regimen, and does not imply any change in the viral pathogenicity, at least in the short period we have investigated.

Death toll in Lombardia during the first months (March–April) of the epidemic shows a “fractal” profile that remains evident even with the natural smoothing due to the summation (Fig. 3D). By no doubt, a non-negligible number of patients died in nursing home residences in Lombardia and, therefore, they were lost from the hospital records. It is likely that this behavior has contributed in “flattening” the ICU curve. However, this bias likely does not hinder the overall profile. We can hypothesize that a mix of factors participate in “compensating” for the potentially distortive effects of deaths occurring outside hospitals, as several casualties—who had tested positive for SARS-CoV-2—died in hospitals for other causes although their deaths were (erroneously) attributed to COVID-19. This finding has been observed in Italy as well as in other countries^18^. Indeed, reliable estimate of Covid-19 fatalities is not an easy task, given that several factors participate in hampering such issue and only the estimate of mortality rate excesses could help us in assessing the true impact of Covid-19. Indeed, as stressed by Chin et al., “models are really only as good as the assumptions that you put into the model. […] how can one expect quality predictions when the data are suspect? Clearly, if the data are suspect, projections may also be sub-optimal”. Precisely, this uncertainty applies for models based on the relationship in between ICU and death rates^19^.

These findings bring some relevant consequences: (a) despite infection and death rates values being untrustworthy, recording of critical care units accesses rates can provide a useful estimate of epidemic dynamics, especially if we aim to appreciate the most relevant medical outcomes; (b) decrease in critical care units accesses rates mirrors the ones we observed for death rates.

Given the reduction in hospitalization in intensive care structures is mostly ascribed to improved efficiency of medical treatments delivered at home or during standard hospitalization, it can be reliably surmised that early and efficient pharmacological therapy can successfully reduce both accesses to critical care units as well as death rates. While the reasons behind the (apparent) high incidence of fatality rates in Lombardia still remain uncovered, we can exclude that differences in virus pathogenicity could provide an affordable answer. The impressive trend we observed in Lombardia from the outset (end of February 2020) simply demonstrated that, since the beginning, the number of (asymptomatic) infected individuals reached a “critical” mass that ultimately caused a sudden “eruption”. Indeed, almost half of the COVID-19 cases registered in Italy occurred in Lombardia.

Figure 4 clarifies both the possibility to follow the epidemic relying only on the number of fatalities (due to ICUs-Fatalities correlation the same result can be achieved by substituting deaths with ICUs) and the qualitative difference between the first epidemic cycle and the so called ‘second wave’ relative to August–September period. It is evident from that picture the possibility to locate the peak (20 April) and the end (around 16–20 June) of the epidemic cycle in terms of the joint increasing/decreasing phase of the infected/dead variables (vector points correspond to subsequent days). Starting from July, we observe the decoupling of dead number from the epidemic cycle with an increasing number of infected cases (trajectories goes right on the x axis) without a relevant increase in cumulative dead count.

In our opinion, this phenomenon is due to the prevalence of asymptomatic cases in the July–September phase discovered by extensive performance of swab analyses, executed especially on young and healthy people, while in the first epidemic cycle the analyses were biased toward more aged, symptomatic, and potentially fragile persons.

The Verhulst-Pearle sigmoid model allowed us to reconstruct the time and intensity of the epidemic peak and to highlight the “hidden” initial period of the epidemic (backward prediction). Namely, we have been able in dating back the beginning of epidemic some months before the first ascertained case. This occurrence, that has been acknowledged by recent investigations^20^, tells about an ‘*hidden circulation*’* of the virus in Lombardia long ago before any official evidence* that could be one of the concurring causes of Lombardia singularity. On a more methodological ground, it is worth noting how the sigmoidal model was able to correctly estimate the time course of the derivative of epidemic spread (the same information conveyed by SEIR-based R-like statistics) when fed of only early (and very uncertain) data before 24 March 2020 .

## Concluding remarks

As aptly remarked^4^, R-based statistics in many cases provide imprecise estimates of epidemic dynamics that both rest on unverified assumptions and do not capture the current status of an epidemic spiking up and down when case numbers are low. Moreover, they are out of scope in situations with marked spatial heterogeneity. It is now widely accepted the ‘over-dispersion’ of R-like indexes^21^ with the great majority of cases due to the so called ‘super-spreaders’ accounting for around 80% of contagion that thus happens in “clusters”. This condition makes ‘average transmission rate’ indexes largely ineffective for predicting the epidemic dynamics.

Other measures (trends in new infections rate, deaths and hospital admissions, cohort surveys) could provide a more reliable estimate of how many people in a population currently have the disease (or have had it). Overall, R index is “too large, and is being used for purposes for which it was never intended”^4^. Here we set some feasible alternatives fixing the above fallacies by checking some of the above-mentioned^4^ alternative strategies.

Indeterminacy of the estimate of affected people, by means of a general underestimation of the asymptomatic pool, might have biased the models on which official statistics usually rely. Furthermore, the fact that data on the infection spreading have been mostly obtained through Real-Time RT-PCR diagnostic—a tool burdened by a huge rate of unreliable results^22,23^—is an additional source of uncertainty. It is noteworthy that serological-based surveys have evidenced incidence rates 40–80-fold higher than those suggested by previous estimates, based only on RT-PCR^24^. Another source of bias is linked to the fact that the beginning of the epidemic has been overlooked by missing a non-negligible number of affected patients (especially in the time lapse of November 2019 –January 2020). Tracing back the early starting point of the Sars-CoV-2 outbreak is a difficult task, besides some attempts have been done in that field^1^; our results suggest a sensible way to achieve this goal by means of sigmoidal models much less demanding than classical SEIR ones.

The tracing back the beginning of the epidemic to October 2019. In addition to genetic distance analyses^17^, indirect evidence has been recently provided by mass media relating the emergence of “strange” interstitial pneumonitis during the fall and evidence is mounting that COVID-19 spreading should be backdated also in other countries, including China. This reconstruction casts more than a shadow upon the current narrative and requires an in-depth investigation of the true origins of COVID-19 epidemic.

Uncertainty affects even lethality rate estimation. Lethality (or Case Fatality Ratio, CFR) is a measure critically dependent on the accuracy of incidence estimation. It is commonplace to explain differences recorded in fatality rate registered in different countries by advocating differences in demographic structure or in the efficiency of public health systems. Indeed, Italy has the most elderly population in Europe and the second most elderly population in the world after Japan. COVID-19 has a strong age dependence for the severity of the infection and the risk of death. However, lethality rate in Japan is far lower (3.2%) than that recorded in Italy (8,8%, October 2020)^25^, the similarities in between the two countries notwithstanding. Moreover, the overall burden of casualties ascribed to Sars-CoV-2 might be easily flawed by the choice of exclusion/inclusion criteria. Indeed, it is difficult to differentiate between deaths in which COVID-19 is detectable from deaths caused by SARS-CoV-2 infection because the vast majority of patients who died in Italy had one or more major pathologies (98.8% with at least 1 comorbidity, and 48.6% having 3 or more diseases) that contributed to the death^26^. Additionally, the death trend also reflects several intertwined factors and represents the ultimate terminus of a complex process that started much earlier. Therefore, the simple daily death recording unlikely could provide a significant picture of the epidemic dynamics. Overall, these factors are among those that contributed to undermine the effectiveness of epidemic management^27^. Indeed, in “the absence of prevalence and incidence data, including the results of serology testing, it is difficult to predict the effects of specific major public health decisions”^28^.

To derive easy to grasp, but reliable estimates of the time course of epidemic, here we propose the use of a new index (RI) that allows to detect tipping point (and then the change of pace of epidemic going to the end of its cycle) in a reliable way.

At the same time, the relation between fully recovered individuals and deaths could represent a trustworthy and unbiased estimator of the epidemic process. It is worth of interest that, when the number of accesses is plotted against the number of COVID-19-related casualties, we found a near to unity relation between these two parameters largely invariant in time and space (i.e., Italian regions display the same trend without appreciable differences).

This strong and invariant relation makes trends of care unit’s occupancy predictive of epidemic extinction, with accuracy higher than that offered by rate of infected or recovering patients, which are biased by uncertainty of collected data and regional-related fluctuations.

### Epidemiological data

The repository from where data were obtained comes from the Italian ministry of Health (http://www.protezionecivile.gov.it/home) and the open data repository can be found at http://opendatadpc.maps.arcgis.com/apps/opsdashboard/index.html#/b0c68bce2cce478eaac82fe38d4138b1.
